# Applications of Large Language Models in the Field of Suicide Prevention: Scoping Review

**DOI:** 10.2196/63126

**Published:** 2025-01-23

**Authors:** Glenn Holmes, Biya Tang, Sunil Gupta, Svetha Venkatesh, Helen Christensen, Alexis Whitton

**Affiliations:** 1 Black Dog Institute University of New South Wales, Sydney Randwick Australia; 2 Applied Artificial Intelligence Institute Deakin University Melbourne Australia

**Keywords:** suicide, suicide prevention, large language model, self-harm, artificial intelligence, AI, PRISMA

## Abstract

**Background:**

Prevention of suicide is a global health priority. Approximately 800,000 individuals die by suicide yearly, and for every suicide death, there are another 20 estimated suicide attempts. Large language models (LLMs) hold the potential to enhance scalable, accessible, and affordable digital services for suicide prevention and self-harm interventions. However, their use also raises clinical and ethical questions that require careful consideration.

**Objective:**

This scoping review aims to identify emergent trends in LLM applications in the field of suicide prevention and self-harm research. In addition, it summarizes key clinical and ethical considerations relevant to this nascent area of research.

**Methods:**

Searches were conducted in 4 databases (PsycINFO, Embase, PubMed, and IEEE Xplore) in February 2024. Eligible studies described the application of LLMs for suicide or self-harm prevention, detection, or management. English-language peer-reviewed articles and conference proceedings were included, without date restrictions. Narrative synthesis was used to synthesize study characteristics, objectives, models, data sources, proposed clinical applications, and ethical considerations. This review adhered to the PRISMA-ScR (Preferred Reporting Items for Systematic Reviews and Meta-Analyses extension for Scoping Reviews) standards.

**Results:**

Of the 533 studies identified, 36 (6.8%) met the inclusion criteria. An additional 7 studies were identified through citation chaining, resulting in 43 studies for review. The studies showed a bifurcation of publication fields, with varying publication norms between computer science and mental health. While most of the studies (33/43, 77%) focused on identifying suicide risk, newer applications leveraging generative functions (eg, support, education, and training) are emerging. Social media was the most common source of LLM training data. Bidirectional Encoder Representations from Transformers (BERT) was the predominant model used, although generative pretrained transformers (GPTs) featured prominently in generative applications. Clinical LLM applications were reported in 60% (26/43) of the studies, often for suicide risk detection or as clinical assistance tools. Ethical considerations were reported in 33% (14/43) of the studies, with privacy, confidentiality, and consent strongly represented.

**Conclusions:**

This evolving research area, bridging computer science and mental health, demands a multidisciplinary approach. While open access models and datasets will likely shape the field of suicide prevention, documenting their limitations and potential biases is crucial. High-quality training data are essential for refining these models and mitigating unwanted biases. Policies that address ethical concerns—particularly those related to privacy and security when using social media data—are imperative. Limitations include high variability across disciplines in how LLMs and study methodology are reported. The emergence of generative artificial intelligence signals a shift in approach, particularly in applications related to care, support, and education, such as improved crisis care and gatekeeper training methods, clinician copilot models, and improved educational practices. Ongoing human oversight—through human-in-the-loop testing or expert external validation—is essential for responsible development and use.

**Trial Registration:**

OSF Registries osf.io/nckq7; https://osf.io/nckq7

## Introduction

### Background

Prevention of suicide is a global health priority [1]. Approximately 800,000 individuals die by suicide yearly, and for every suicide death, there are another 20 estimated suicide attempts [1]. Despite the largely preventable nature of suicide, factors such as limited service capacity, variable service quality, and barriers to service access significantly impact the progress being made toward reducing suicide rates [2]. Recent advances in transformer-based artificial intelligence (AI)—the technology that has accelerated the development of powerful large language models (LLMs) that human clinicians and consumers can converse with—have been suggested as a potential solution to enhancing the scalability, accessibility, and personalization of health care interventions [3]. In the context of suicide research and care, LLMs can generate novel insights into suicide risk by parsing language, classifying or scoring text, and comprehending and generating human-like language, with the potential to enhance treatments by improving user engagement with digital interventions. Such improvements can also expand the capacity of crisis support services by providing real-time crisis clinician copilots, automated risk assessments, or expedited triage, enabling the optimized use of human resources. In addition, LLMs can improve the quality of training programs for those who may intervene by mentoring trainees through clinical scenarios and flexibly adopting a variety of crisis-seeking personas for clinical role-play scenarios. Hence, LLM technology might be a critical catalyst for advancement in the field. However, the relatively unexplored nature of the field means that we are only just beginning to understand how LLMs can be harnessed safely and effectively for suicide prevention. In parallel, there is a pressing need to explore how emerging trends in LLM-based research, such as the nature of training data, model types, the contexts of application, and methodological norms from relevant research fields, may shape the trajectory and direction of suicide prevention research (either positively or negatively), now and into the future.

AI refers broadly to machine and computational processes used to execute tasks usually thought of as requiring human intelligence. Natural language processing, a language-focused subfield of AI, has advanced significantly with the advent of transformer-based LLMs [4]. LLMs are the current state-of-the-art technology in computational linguistics, comprising attention-based language encoders trained using massive text datasets, generally with billions of parameters [5]. In addition to language comprehension, LLMs have recently shown their utility in generative language applications, such as generative AI interfaces (chatbots) [6]. The availability of LLMs, such as OpenAI’s generative pretrained transformer (GPT) models, Google’s Bard, and Meta’s Llama, has created unprecedented opportunities for language generation and analysis at scale [7], with LLM applications already becoming widespread across fields such as business analytics, commerce, administration, and education [8].

Under the right conditions, LLMs can demonstrate contextual understanding and content generation that closely mimics human interaction. Accordingly, research into LLM applications in health care settings, where human interaction forms the basis of much of service delivery, is accelerating at a rapid pace; for example, LLMs have been investigated as a potential aid in preconsultation, diagnosis, and the management of disease (eg, infectious diseases and cancer [9-11]), as well as in recommending specialist appointments via SMS text messaging–based self-assessment tools for remote populations [12] and generating patient education materials [13].

Given that language is the primary basis upon which symptoms are reported and assessed in mental health, LLMs represent a significant technological advancement in mental health research. LLMs are currently under investigation for their utility in cognitive behavioral therapy facilitation [14], for emotion identification during psychotherapy sessions [15], and for the detection of positive therapeutic behaviors during motivational interviewing [16]. LLMs have also shown potential benefits in helping individuals understand personal coping styles and in facilitating stress reappraisal [7]. The application of LLMs in the field of suicide prevention is particularly promising, given that traditional methodologies, which have struggled to provide actionable insights into complex constructs such as suicide risk, are complemented by the analytic and generative capabilities of LLMs [17].

In evaluating the promising aspects of LLMs, it is also important to consider that these models present a number of challenges to researchers and end users alike. LLMs are often criticized for the inaccuracy of generated information, reducing trust and credibility [5], a failing that is compounded by the lack of interpretability of LLM functioning resulting from their “black box” architecture. Challenges also exist around data security and privacy; for example, LLMs have been demonstrated to identify individuals from deidentified digital data (eg, location data, medical billing information, or a collection of social media posts and metadata) [17]. When considering application contexts, the generalizability of LLMs is restricted to the degree of representativeness of the training data with respect to age, ethnicity, or even those who use social media versus those who do not [18]. Nevertheless, LLMs hold promise for improving models of suicide risk (eg, via digital phenotyping) [17]; for creating synthetic data [19] to increase sample sizes and statistical power for research on low base-rate events such as suicide; for powering conversational agents that support early triage, crisis support, and help seeking; and for clinician assistance tools, such as automated patient assessment systems, and simulation of crisis scenarios to improve training outcomes. Despite these potentialities, the integration of LLMs into suicide prevention currently trails other fields. Critically, significant gaps must be addressed for the field to move forward in a manner that is safe and acceptable, including understanding the interpretability of LLM outputs, addressing biases within training data, and ensuring ethical deployment within prospective crisis settings.

To date, there have been no reviews examining the integration of LLMs into research on suicide prevention and self-harm. Prior reviews have focused predominantly on research undertaken in adjacent fields, such as applications of machine learning or chatbots to mental health more broadly [20-23]. Although several reviews have explored the use of machine learning [24-27] or AI-based strategies [28-31] in suicide prevention contexts, these have not focused on applications of LLMs specifically. Furthermore, although 1 commentary [32] and a review [25] focused on the integration of computational linguistics or natural language processing more broadly in suicide prevention, these articles did not focus on LLMs specifically, nor have any explored applications of LLMs to self-harm.

### Objectives

The aim of this scoping review was to provide an understanding of current applications of LLMs in the field of suicide prevention, including applications to self-harm. The research questions guiding this review were as follows:

1. What trends are present in the literature with regard to the models used, data sources, and LLM objectives?
2. What are the clinical applications reported in the literature?
3. What ethical considerations are noted in the literature?

Specifically, we aimed to identify emerging trends in the literature related to the types of training data, models used, and intended objectives.

Our secondary aim was to explore reported potential clinical applications of LLMs (eg, as clinical copilots or to augment suicide prevention training programs) and identify ethical considerations that are crucial as the field progresses. Reported potential clinical applications were sought to better understand the future trajectory of practical LLM use in the field, with ethical considerations identified to understand the important considerations for practical deployment.

A *scoping* review was selected to map the breadth of evidence across the varied fields from which LLM-based research originates to identify key characteristics of studies, reveal knowledge gaps, and inform future research [33]. In doing so, we aim to highlight emergent trends in the research and address critical clinical and ethical considerations to ensure safe, effective, and equitable research outcomes in this key field.

## Methods

This review is presented in line with the PRISMA-ScR (Preferred Reporting Items for Systematic Reviews and Meta-Analyses extension for Scoping Reviews) checklist (Multimedia Appendix 1) and was developed and carried out with reference to the JBI best practice methodological standards [34,35]. In accordance with the JBI recommendations [36], the protocol was preregistered on the Open Science Framework and is publicly available [37].

### Inclusion and Exclusion Criteria

To meet the eligibility criteria, studies needed to describe the application of an LLM to the area of suicide or self-harm. Although various natural language processing models using machine learning methods exist (eg, support vector machines, Bayesian networks, and random forest algorithms), for this review, we focused on contemporary LLMs, defined as computer-engineered language models that use transformer-based neural network architecture [4]. Although LLM training parameters generally exceed 10 billion, there is no formal consensus on parameter scale [6]. In this review, smaller models (<10 billion parameters) such as early incarnations of the Bidirectional Encoder Representations from Transformers (BERT) and Text-to-Text Transfer Transformer (T5) were eligible for inclusion as they represent language models capable of contextual understanding. Studies describing the use of AI, machine learning, or big data approaches in the absence of LLM methodology were ineligible.

The domain of suicide prevention included, but was not restricted to, areas such as ideation, planning, attempts, prediction, intervention, support, means restriction, gatekeeper training, and public awareness campaigns, as well as self-harm. Studies focusing on LLM applications to mental health or psychiatry that did not specifically mention suicide or self-harm were ineligible. Studies describing quantitative, qualitative, or mixed methods designs were eligible. All studies involving human participants were required to report ethics approval. Source documents were peer-reviewed journal articles or conference proceedings. Conference proceedings were included as they have become the dominant form of published research in computer science (encompassing LLMs) in recent years [38]. Conference proceedings were required to have a comprehensive methodology and results sufficient for replication. Conference abstracts were excluded, as were other reviews and meta-analyses.

Studies using electronic health records (EHRs) were not included in this review. EHRs represent a specific type of corpus representative of individuals who are in contact with the health system. Given that a significant proportion of individuals experiencing suicidal ideation are not in contact with formal health services [39], models that draw primarily on EHR data may generate insights that do not generalize to the broader population of individuals who experience suicidality or self-harm. In addition, substantial work has focused on the use of EHRs for the prediction of suicide risk in recent years [20], with the potential for these studies to populate a stand-alone systematic review. This review sought to elicit an understanding of novel deployments of LLMs, particularly in the individual use context, and to propose future use possibilities or potentialities for support, treatment, or prevention. Studies not published in English were also excluded from this review. There was no limitation on country of origin or publication date; however, the advent of transformer architecture in 2017 naturally limited publications from prior years.

### Search Strategy

Searches were conducted in 4 databases: PsycINFO, Embase, PubMed, and IEEE Xplore. An initial search was conducted on December 6, 2023. Following abstract and full-text screening, the search was updated on February 16, 2024, to ensure recency of the search prior to final text extraction. Additional studies identified via citation chaining were also included at this point.

The search strategy used index terms and free-text terms to cover two core themes: (1) LLMs and (2) suicide or self-harm. Individual database search strings are provided in Table S1 in Multimedia Appendix 2. Search results were imported into Covidence review management software (Veritas Health Innovation Ltd) [40], which was used for abstract and full-text screening as well as data extraction.

### Study Selection and Screening Process

Reflecting prior systematic research [41], title and abstract screening were conducted by 1 author (GH), with a sample of studies reviewed by multiple authors during screening and extraction. In line with recommendations for ensuring reliability and bias mitigation [33], 20.2% (77/381) of the studies identified in the initial search were randomly selected and reviewed by a second author (BT). Agreement was achieved for 71 (92%) of the 77 studies. Conflicts were discussed and resolved with input from a third author (AW).

A similar process was adopted for full-text screening, which was conducted by 1 author (GH), with 20.3% (38/187) of the full texts randomly selected for review by a second author (BT). There was 92% (35/38) agreement between the authors. Conflicts were discussed and resolved with input from a third author (AW).

### Data Extraction

The data extraction template was piloted with 9% (4/43) of the included studies by 2 authors (GH and BT) in line with prior research [41,42], resulting in >90% agreement. Minor refinements were made to the template to assist later synthesis. Data extraction from the 43 included articles was performed by 1 author (GH), with 19% (8/43) of the studies randomly selected and data independently extracted by a second author (BT). The comparison of extracted data showed >90% agreement. The data extraction template included items under the headings of study characteristics, methods, outcomes, and reproducibility and is available in Table S2 in Multimedia Appendix 2.

### Analysis

Studies were classified across deductively derived categories, with categories based on previous research [42] and with scope for adjustments during the review process if required (eg, the aggregation of specific data sources to report on social media more generally as a source of data). Descriptive statistics were computed and presented in textual, tabular, or graphical format, as appropriate. A narrative synthesis of the included studies was conducted to answer the key research questions.

## Results

### Selection of Sources of Evidence

**Figure 1:**
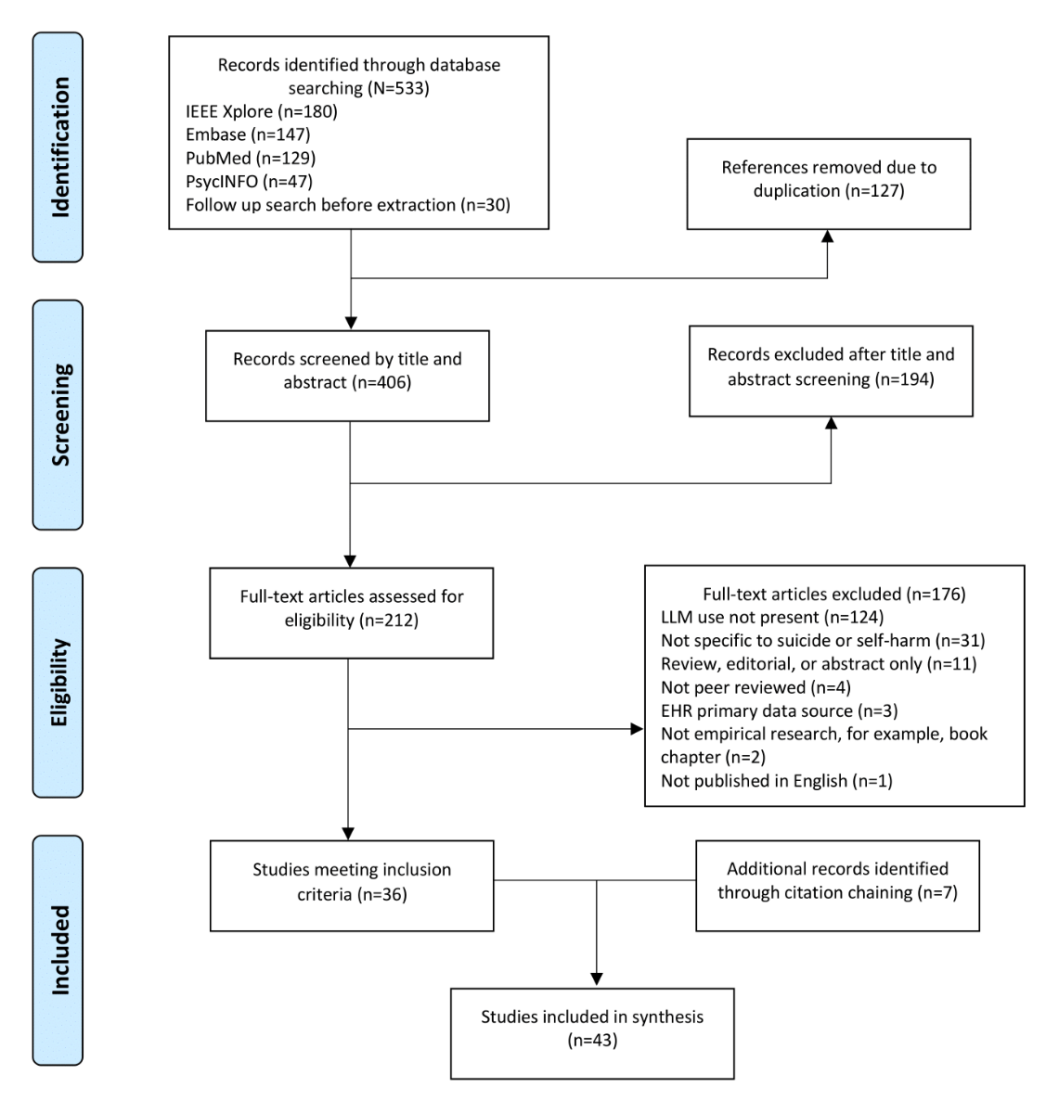
PRISMA (Preferred Reporting Items for Systematic Reviews and Meta-Analyses) flowchart. EHR: electronic health record; LLM: large language model.

The search results are presented in the PRISMA (Preferred Reporting Items for Systematic Reviews and Meta-Analyses) flowchart (Figure 1). The search yielded 533 studies; after removing 127 (23.8%) duplicates, 406 (76.2%) studies were retained for title and abstract screening. Of these 406 studies, 194 (47.8%) were excluded, leaving 212 (52.2%) for full-text review. After full-text screening, 176 (83%) of the 212 studies were excluded (the reasons for exclusion are shown in Figure 1), and 36 (17%) studies met the inclusion criteria. An additional 7 studies were identified through citation chaining, resulting in 43 studies included for synthesis.

### Characteristics of Included Studies

A full list of included publications and associated results data is presented in Multimedia Appendix 3 [43-85]. The number of publications by year demonstrated a compounding pattern, with the first publications emerging in 2019 after the development of the transformer architecture [4] in 2017 and then increasing sharply in 2023 after the release of ChatGPT to the public in November 2022.

The articles identified for this review came from 20 different sources that predominantly came from 2 fields (determined by reviewing individual journal aims and scope): computer engineering (23/43, 53%) and health (18/43, 42%). The bifurcated nature of the publication field of origin demonstrates the cross-disciplinary nature of LLM research. Approximately a third of the studies (16/43, 37%) were published as conference proceedings via IEEE Xplore, reflecting publication norms in computer engineering [38]. The largest proportion of studies came from the United States (10/43, 23%). Study funding was predominantly sourced through grants (23/43, 53%). Of the 43 studies, 4 (9%) indicated no funding, and 16 (37%) did not provide funding information.

### Synthesis of Results

#### Base LLMs Used

**Figure 2:**
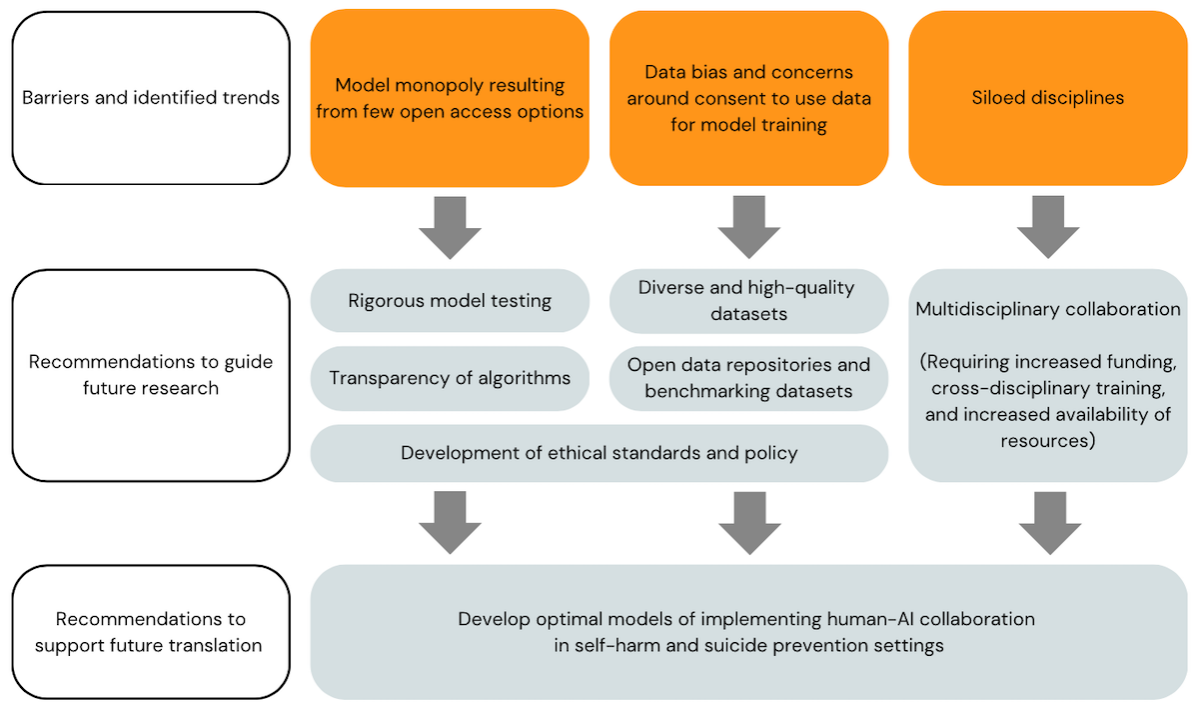
Trends and associated recommendations for ensuring safe and effective integration of large language models into suicide prevention and self-harm research. AI: artificial intelligence.

A synthesis of identified trends with associated recommendations is presented in Figure 2. Most of the studies (35/43, 81%) applied some derivation of Google’s BERT. OpenAI’s GPT family of LLMs were used in 9 (21%) of the 43 studies. Other models included XLNet (3/43, 7%); Google’s Fine-Tuned Language Net (FLAN; 2/43, 5%); and Alpaca, Alexa, DeepMoji, and contrastive language-image pretraining (1/43, 2% each). Some studies applied >1 model type. The widespread adoption of BERT can be attributed in part to its status as one of the earliest open-source models (accessible since its inception in 2018), allowing users to freely download and use the model for research purposes. FLAN [86] is also open source, though more recently released, in 2022. Contrastive language-image pretraining, DeepMoji, Alexa, and GPT-3.5 are accessible via an application programming interface but are not open-source models, limiting data transparency and the reliability of availability required for most research applications.

In 33 (77%) of the 43 studies, the primary purpose of LLMs was contextual understanding of language, while 9 (21%) studies extended upon this by using the capacity of LLMs to generate natural language responses to prompts (all these studies were published in 2023). The study by Badian et al [47] also used an LLM for image interpretation. Applications that focused on contextual language understanding were predominantly used for the identification, detection, or prediction of suicide risk. Generative applications also focused on prediction-based tasks [43,44,83] but extended to the evaluation of suicide risk [58,61,66], the identification of circumstances preceding suicide [85], information retrieval or question-answering systems [46], and the creation of mental health nursing care plans [81]. Of the 9 generative applications, 7 (78%) used the text-based ChatGPT user interface, while the remaining 2 (22%) used text-based interfaces for an educational BERT model [46] and a data-secure FLAN model [85]. The prevalence of GPT use in generative applications stands in contrast to the dominance of the use of the BERT model across LLM-based research to date more generally. This hints at a potential shift away from studies primarily focused on BERT. Whether this trend reflects a broader shift in model preference or was influenced by factors such as availability, the ease of use, or specific application requirements was not clear from the included studies.

#### Data Sources

The majority of the included studies (31/43, 72%) used data from user posts on social media platforms for LLM training, validation, testing, and deployment to answer a research question. Reddit was the most commonly used platform from which data were derived (18/43, 42% studies), followed by X (formerly Twitter; 13/43, 30% studies). User posts from other platforms were also used [51,52,82,84]. Meta’s Facebook was used only in the study by Badian et al [47], in which only images, not text data from users, were used. Of the 31 studies using social media data, only the study by Badian et al [47] sought user or poster consent for the use of their data. Of the 12 studies that did not use social media data, 3 (25%) used data from crisis counseling apps or services [48,59,73]; 6 (50%) used a heterogeneous collection of data from the National Violent Death Report System [80,85], educational or academic documents [46,64], or participant vignettes [66,81]; 1 (8%) used research participant data gathered during the course of the study [70]; 1 (8%) did not use collected data but applied prompt inputs to ChatGPT to evaluate responses [61]; and 1 (8%) proposed an LLM-moderated train safety device, designed to initiate braking and deploy an inflatable safety cell in front of the train after voice activation by the train driver [77].

#### Identified Objectives

Studies were grouped by 4 main objectives: prediction, identification or classification, support, and education or training. The majority of the studies (33/43, 77%) identified in this review applied LLM models to identification or classification tasks, with prediction applications the next most common (6/43, 14%). Studies that focused on the problems of identification or classification sought to identify content indicative of suicidal distress from text-based data, such as in Reddit posts [49,56-58,60,63,68,71,76], in X (formerly Twitter) posts [45,53,69,72,78], or in crisis or helpline conversations [48]. Additional uses included identifying precipitating events to suicide from death investigation narratives [80] and identifying self-harm from social media posts [54]. Prediction-focused studies predominantly used LLMs with data from Reddit [50,65,83], other social media [84], images posted on social media [47], crisis counselling data [59], or clinical data [50] to predict suicide risk.

The remaining model applications (4/43, 9%) assessed support [61], education [46], and “other” (ie, the generation of mental health care plans and means restriction) [77,81]. Of these 4 studies, 3 (75%) involved the use of generative AI. Regarding education, a question-and-answer interface was developed to generate specific information requested by individuals at risk and their families. The model underlying this interface drew on a corpus of >300 suicide-specific documents curated by clinicians [46]. Of the 2 studies that involved ChatGPT, 1 (50%) aimed to assess the safety of publicly available conversational agents by prompting them with sequential Patient Health Questionnaire-9 items to examine chatbot responses to a patient simulation indicating escalating suicide risk [61], while 1 (50%) asked ChatGPT to generate mental health nurse care plans based on vignettes about a fictitious person self-harming [81]. Overall, study objectives focused on suicide and self-harm detection, while the development of generative LLM technologies has facilitated more recent LLM use in care, support, and education.

#### Clinical Applications

We examined trends in proposed clinical applications of LLMs to suicide or self-harm prevention. Studies were expected to have provided sufficient depth of discussion regarding ways in which the research could be translated into real-world settings. Brief general statements or single-sentence phrases about clinical applications were not considered sufficient for inclusion in addressing this research question.

Clinical applications were discussed in 26 (60%) of the 43 studies. Of the 17 publications that did not discuss clinical applications, 13 (76%) were from the field of computer science. The identified applications included improved detection of suicidality (13/26, 50%), use as a clinical assistance tool (10/26, 38%), improved accessibility of services (3/26, 12%), improved services (3/26, 12%), assisting the development of policy (3/26, 12%), and use as a training tool (2/26, 8%). Specifically, studies discussing the ability of LLMs to improve the detection of suicidality mentioned the creation of automated systems that could be applied to language data (eg, social media posts) to detect suicidal ideation for early intervention [48,55,57]. Studies focusing on the potential applications of LLMs as clinical assistance tools discussed possible use cases where LLMs could aid clinicians in evaluating a client’s level of suicide risk [48,57], supporting diagnosis and treatment [50,67], providing a second opinion [66], or predicting a score on a mental health scale [83]. Future-oriented clinical assistance tool applications included AI-enabled avatars that could be accessed remotely, could deliver therapeutic services, could conduct simple examinations, provide advice, or recommend referrals for additional care [66]. Applications related to service improvement included LLM integration to improve crisis counseling services [48,73] and improving existing mental health chatbots [54]. Applications to training included enhancing training, clinical procedures, and best practices among mental health and medical professionals [73,79]. Improved policy was noted as of clinical relevance [73,79] because there may be a lack of policy safeguarding people considered vulnerable and the use of LLMs [81]. Cost-effectiveness and increasing the quality of data annotation were also noted [58], as was use in public health surveillance, potentially allowing practitioners to track the prevalence of infrequent conditions [85].

#### Ethical Considerations

We also explored trends in the nature and type of ethical considerations reported. To be considered as having meaningfully reported on ethical issues, studies needed to include discussion of the ethical considerations and their possible implications. Cursory mention of potential ethical issues without discussion of their implications was not considered sufficient (eg, mention of LLMs as being a “black box” without any further discussion of the implications [47,59] was not considered sufficient).

Ethical considerations were discussed in 14 (33%) of the 43 studies. Of the 29 studies not presenting ethical perspectives, 18 (62%) were published in computer engineering journals, where greater emphasis was given to the technical aspects of model development, training, and validation. Privacy (9/14, 64%) was the most common ethical consideration discussed, followed by bias (5/14, 36%), hallucinations (the tendency of LLMs to sometimes generate false responses; 3/14, 21%), the “black box” nature of LLMs (3/14, 21%), possible threats to the client-clinician relationship (eg, clinicians replaced with digital agents; 2/14, 14%), and false positives or negatives in classification or identification (2/14, 14%).

Specifically, studies that discussed ethical issues regarding privacy touched on concerns about confidentiality and consent to use publicly available data [55,75,82,83], with researchers highlighting the need to value the privacy, security, and anonymity of the original posters and end users [57,66]. Some researchers presented ethical concerns related to publicly traded companies inferring or collecting sensitive health information about their users and acting on it or sharing it without explicit consent [50,58]. Nontransparent data collection and inference processes currently used by social media platforms were highlighted as a growing area of concern [74]. Hallucinations were identified as impacting the safety and reliability of LLM applications [58,85]. Some of the studies (2/14, 14%) raised concerns that models trained on vast amounts of data containing social biases or prejudices may perpetuate stigma toward suicide or self-harm [58,83]. Related concerns were raised regarding the generalizability of LLMs to populations whose racial, ethnic, or cultural demographics differ from those present in training data [48,75]. The study by Levkovich and Elyoseph [66] noted that these data biases or the lack of diversity could lead to erroneous predictions that may exacerbate existing disparities in suicide prevention strategies. Furthermore, 3 (21%) of the 14 studies noted a lack of transparency, resulting from the opaque functioning of the underlying algorithms (termed “black box”) [59,66], with the study by Woodnutt et al [81] noting a lack of governance in this regard.

Of the 14 studies, 2 (14%) raised concerns about LLMs threatening the client-clinician relationship, potentially leading to human-centered clinical interactions being replaced by digital alternatives. Karapetian et al [64] discussed the issue of LLM agents lacking human attributes, such as empathy for suicidal distress, and Malhotra et al [67] noted that the integration of LLMs into clinical care may lead to a sense of distancing of the clinician from the individual, potentially fostering feelings of invalidation or insignificance and exacerbating suicidal thoughts or self-harm behaviors. False negatives (when suicidality goes undetected) and false positives (when suicidality is incorrectly flagged as being present) were noted as concerns [74,75] as psychological harm can result (eg, resulting in missed opportunities to intervene with someone at risk or in unnecessary mental health evaluations for someone who is not at risk) [74]. Safety was also noted as a concern because conversational AI may advance at a pace that outstrips associated safety measures [61]. Relatedly, issues of clinical responsibility were highlighted, particularly regarding the use of LLMs in aiding the generation of mental health care plans, as some authors believed that this could leave mental health practitioners legally vulnerable [81]. More generally, studies emphasized that guidelines for safe development, auditing, and regulation are very much needed to address ethical risks in this area of research [83].

## Discussion

The primary aim of this scoping review was to summarize and characterize emerging trends in the application of LLMs in the field of suicide prevention and self-harm research. This review maps the study characteristics and key methodological components of the included studies (n=43) and further addresses the secondary aims of examining the clinical applications and ethical considerations proposed in the included studies.

### Study Characteristics

The studies included in this analysis exhibited a notable divergence in disciplinary focus, with approximately equal representation from computer engineering (23/43, 53%) and health-related (18/43, 42%) fields. The bifurcation of domain expertise in this emerging field has important implications for the safety, effectiveness, and real-world impact of the research outputs, particularly considering the differences in technical training, publication norms, and approach to validation and evaluation used across the different fields; for example, only recently has there been a call to integrate education in the technicalities and ethics of AI into mental health training programs, and many mental health researchers may not yet receive technical training in AI to be able to develop and apply LLMs for clinical research purposes. In parallel, while computer science researchers have significant technical training to enable the development of innovative AI models and access to industry partnerships that can support the scalability of new AI-based tools, the real-world usefulness of these innovations hinges on a deep understanding of the ethical and clinical context in which they are to be applied, the facilitators and barriers to their use, and the needs and priorities of end users—areas of expertise where clinical training is often paramount. Recognizing and addressing the complementary strengths and gaps in these divergent disciplines will be crucial for ensuring that LLM innovations in suicide prevention are grounded in technical excellence and clinical acumen.

Similarly, the dissemination of research from computer science often outpaces the dissemination of research from the field of mental health owing to the stronger focus on conference proceedings with expeditious publication timelines. The accelerated dissemination of LLM-based suicide prevention research originating from computer science fields may mean that practices focused on innovation—a crucial benchmark of research impact in computer science (eg, via patents) [87]—come to dominate early research advancements in this area [88]. Although innovation is crucial for achieving technological advancements, this innovation often comes at the expense of comprehensive clinical validation [89], and there has been growing emphasis on the need for frameworks for validating new AI tools in health research [90].

Recognizing and navigating these disciplinary disparities is essential. Encouraging multidisciplinary collaboration among experts from diverse backgrounds is likely to be critical for bolstering the quality, safety, and impact of research on LLMs for suicide prevention. This could be done by fostering the development of agile methods for disseminating validation research (such as through cross-disciplinary repositories and benchmarking datasets), establishing sound ethical and safety frameworks that cut across key disciplines (such as living guidelines), and facilitating ongoing education and cross-disciplinary training for researchers across both fields. Crucially, prioritizing and supporting studies led by multidisciplinary teams that rigorously assess safety and effectiveness is imperative for ensuring that research efforts lead to broader societal benefits.

### Base LLMs Used

There was minimal variation in the base LLM model applied in the identified studies. Most of the studies (35/43, 81%) used either the base BERT model or some variation of a trained or fine-tuned BERT model [91]. The relatively early release date (2018), open-source availability, compact size of this model compared to subsequent models such as GPT, and its adaptability through fine-tuning for diverse applications are all factors assisting the widespread use of BERT in this research area. While open access is critical for facilitating replication and democratizing access to this modern technology, an important consideration is that open-access LLMs trained on generalized datasets might inadvertently perpetuate unwanted biases; for example, BERT has been shown to exhibit stereotypical biases in areas such as gender, profession, race, and religion, with this bias not related to the size of pretraining corpora but likely due to the nature of the training data [92]. In the same study, GPT-2 was found to have less bias, hypothesized in part to be due to antistereotypical associations present in the training data. LLMs are “stochastic parrots” [93] in their generative responses, their outcomes dependent on the quality of the input training data. Therefore, the testing, application, and comparison of multiple models is important to detect and mitigate model biases permeating an entire discipline. Promoting the use of a diverse range of models to assess performance variability [58,83] and uncover potential biases [92] could enhance the strength of research outcomes in suicide prevention. In addition, there is a pressing need to develop specialized open-access models (such as those being developed in medicine) [94,95] for mental health and suicide prevention. These models should draw upon the expertise of mental health professionals, cross-disciplinary researchers, ethicists, and individuals with lived experience. Establishing standards and policies that ensure the transparent reporting of model training data and inherent potential biases is also imperative to enable the critical evaluation and validation of model suitability [7], particularly for self-harm and suicide prevention contexts.

### Data Sources

Most of the studies (31/43, 72%) used data from social media platforms such as X (formerly Twitter) and Reddit to train LLMs. Although these are rich data sources, a reliance on social media data as a form of training data may lead to limitations in the usefulness of LLMs. To protect their users, social media platforms often implement policies to detect, suppress, or remove content related to suicide or self-harm. Therefore, using social media data for LLM training may result in an underrepresentation of critical data that would enable LLMs to effectively recognize and respond to expressions of suicidal distress. In addition, algorithms embedded within social media platforms are designed to promote engaging content. As a result, these algorithms often promote user posts that are biased toward certain viewpoints and controversial or sensational topics, while simultaneously minimizing diversity of perspective. Accordingly, the algorithms in social media platforms may drive artificial patterns in the nature and frequency of certain types of user post data that do not reflect real-world interpersonal interactions.

It is also crucial to evaluate whether the profiles and behavior of individuals who post on social media platforms align well with those of the potential end users of LLM-based suicide prevention interventions; for example, while Reddit’s user anonymity may constitute a perceived benefit to users, evidence indicates that users engage in differing behavior when posting anonymously compared to when their identity is known [96]. To mitigate this, it is imperative to curate training datasets that are diverse and balanced [97], as well as representative of the population that the LLM is intended to serve [7]. The inclusion of input from those with lived experience is important during the development of these datasets because these experiences can provide valid human-generated data for model training. Furthermore, lived experience can provide a valuable perspective in reviewing LLM-generated data, enabling key human-in-the-loop validation of otherwise unavailable data for model training (eg, crisis care transcripts). In addition, actively seeking out content, including positive interactions, supportive conversations, and safe discussions around self-harm and suicide prevention, may help enhance the effectiveness of LLM-based suicide prevention interventions.

### Identified Objectives

The studies included in this review predominantly deployed LLMs for the identification, detection, classification, and prediction of suicide or self-harm risk. These areas represent a narrow band of potential use, and despite research focused in this area, there remains a lack of solutions that implement these models in clinical settings [55]. One reason for this is that computer scientists, who are at the forefront of technological innovation in this area, do not generally have in-depth knowledge of clinical processes and workflows used in the mental health field. This limits their ability to envision the broader applicability of LLMs in mental health settings and results in a tendency to gravitate toward more familiar applications, such as classification and regression. Similarly, suicide prevention researchers often do not possess the technical expertise required to adapt LLMs for their specific needs. Consequently, multidisciplinary collaborations are critical to the development of sound research programs that combine the clinical and technical expertise necessary for high-caliber research. As this research advances and models continue to improve, those focused on clinical areas may be deployed in various contexts. However, it has been noted that this may be insufficient and may not reduce suicide attempts or deaths unless the treatment needs of people identified as being at risk are met [98]. Hence, it is imperative to explore innovative solutions in clinical care, consultation, and therapy.

This review highlighted a small number of recent applications that move beyond identification and prediction by leveraging the generative features of LLMs. These applications included assessing whether LLMs could provide access to suicide-related educational information [46], whether they could detect escalating suicide risk and the need for human intervention [61], and whether they could assist in generating mental health nurse care plans for individuals who self-harm [81]. These studies showed that, at present, LLMs can perform well on specific elements of these roles, including providing immediate access to accurate information relevant to suicide prevention [46], using health theory frameworks to construct detailed care plans with tangible goals for clients [81], and generating training aids for mental health providers [81]. However, these studies also highlighted areas where LLMs currently fall short. Specifically, they showed that LLMs did not effectively detect and respond to signs of escalating clinical risk by referring into care [61]. Moreover, concerns were noted regarding how the accountability of health care providers may be impacted by the use of AI-generated mental health care plans [81]. These examples highlight the critical role of human expertise in the deployment of LLMs in suicide prevention contexts, and an important area for future research will be to determine optimal approaches to implementing human-AI collaboration in suicide prevention settings, including through the use of copilot or human-in-the-loop systems that are beginning to be explored in other areas [89,99].

### Clinical Applications

The secondary aim of this scoping review was to consider the clinical applications and ethical considerations of LLMs in suicide prevention practice. Among the studies describing clinical applications of LLMs, “enhanced detection of suicidality” and use as a “clinical assistance tool” were the most commonly presented clinical applications. The ability to identify and predict suicide has remained at near-chance levels for many years [24], and improvement upon this with the help of novel technologies such as LLMs can provide a valuable contribution to the field. However, accurate risk detection alone is insufficient and must be paired with effective and scalable interventions [24]. The use of LLMs as clinical assistance tools can support clinical professionals by aiding with a myriad of tasks [48,57,66,70]. The conception and design of these applications should start with the end point in mind to best plan for the translation of research results into implementation in a clinical setting. Critically, researchers should engage clinicians, health professionals, lived experience representatives, and other relevant stakeholders early in the research project development to ensure that the research can meet the needs of end users. In the case of lived experience representatives, it is imperative to understand how they, as representatives of end users, interact with the various potential applications of LLMs to ensure safety and effectiveness. Clinical applications described in the studies include assisting clinical decision-making by providing a second opinion [66], functioning as a clinician copilot [48], serving as a mental health triage tool [57], and supporting therapy delivery [70]. However, these applications were limited to hypothesized future uses [48,57,66] or pilot studies [70]. More investment is required to develop implementable clinical assistance tools, particularly in supporting the integration of multidisciplinary teams to collaborate on areas such as clinical training and support as well as identifying novel use cases with the potential to positively impact suicide prevention practice. Importantly, LLMs should not replace clinical judgment but rather supplement it, aiding clinicians and health professionals to make more informed decisions [66] by enhancing access to information (eg, suggesting differential diagnoses, proposing alternate hypotheses, or collating scattered information into more coherent forms to facilitate human interpretation). LLM applications should not exceed this human-in-the-loop support-based role until their safety and effectiveness can be clearly demonstrated.

It is also important to consider the context in which an LLM-based application will be implemented, acknowledging that its success may be limited by existing clinical workflows [100] and the overall capacity of the health system to integrate new tools and provide relevant training [101]. As with all new technological advancements in care, LLM integration into health care practices requires strategic integration into existing—often outdated and practically constrained—care models if we are to realize their full benefit. Forward thinking and responsive policy is required, led by governments that engage with stakeholders to map policy and standards to provide for the harmonious integration of LLM clinical support tools, given the accelerating use and potential of AI.

Research indicates that a majority of individuals do not engage with formal mental health services before suicide, potentially due to stigma or fear of judgment [39]. While none of the identified studies deployed LLMs to directly support or intervene with individuals in distress, the ease of access that a language-based user interface offers may help some overcome stigma-related barriers to engaging with formal mental health services. Indeed, preliminary evidence for the acceptability of generative LLMs in support contexts has been demonstrated; for example, evidence shows that a large majority of individuals (78%) would be willing to use ChatGPT for self-diagnosis or to aid in self-managing their symptoms [102]. Some individuals also indicate a willingness to disclose information to nonhuman agents citing reduced fear, less need for impression management, and greater ease in expressing the severity of their emotions [103].

This review found evidence that LLMs can deliver accurate information relevant to suicide prevention [46] and support clinical needs in related contexts such as mental health evaluations, therapeutic consultations, and patient education [104]. Therefore, LLMs hold the potential to offer individuals quick, empathic responses to mental health queries [105], while avoiding potential or perceived judgment and stigma, at little to no cost, available 24/7. Nevertheless, the efficacy of LLMs in providing precise, evidence-based support to individuals in suicidal distress has not been empirically validated. Furthermore, LLMs currently lack the ability to collect information vital for diagnosing and managing a patient’s health condition [106]; for example, ChatGPT’s performance has been found to deteriorate as the complexity of clinical cases increases [105], and research identified in this review demonstrates that GPT-powered conversational agents and chatbots can be dangerously slow to escalate high-risk mental health situations for human clinical intervention [61]. While there may be a safe use threshold at which LLMs are beneficial for information and psychoeducational purposes, they are not advanced enough to be used as stand-alone therapeutic devices. Looking forward, models of LLM use in crisis support contexts are most likely to be those that are driven by clinical oversight, with sufficient guardrails in place to trigger referrals when clinically indicated.

The incorporation of LLMs into training and educational contexts presents a promising opportunity for research and development under clinical oversight. Educational resources, such as the question-answering system in an included study that was aimed at providing suicide prevention information [46], provide an illustrative example. This mode of education is highly scalable and may provide a cost-effective means for deploying training programs. Such educational tools would be particularly useful in large-scale, multifaceted, community-wide interventions such as the European Alliance Against Depression [107] and Lifespan [108] models, both of which have community awareness and training as key components. Another study identified in this review [81] suggests that LLMs such as ChatGPT could support training by assisting less experienced mental health practitioners in creating care plans, brainstorming ideas, or pinpointing relevant aspects of patient presentation. However, it was noted that these generated plans, while appearing credible to laypersons, have been found to contain significant errors and ethical issues upon professional evaluation, highlighting the indispensable role of clinical oversight [81]. LLMs could also facilitate the creation of diverse case scenarios for health care providers to hone their risk assessment and communication skills. By role-playing case scenarios, LLMs can offer real-time, interactive training experiences for crisis call center staff, counselors, and suicide prevention gatekeepers, enhancing their confidence in discussing suicide and building clinical competencies. In addition, the use of LLMs to converse in a role-play scenario has the potential to improve initial training outcomes [109]. Critically, suicide prevention gatekeeper training outcomes are shown to diminish over time [110]; however, engagement with a role-playing LLM could result in improved retention of training outcomes and more individuals at risk identified, approached, and referred for help.

**Figure 3:**
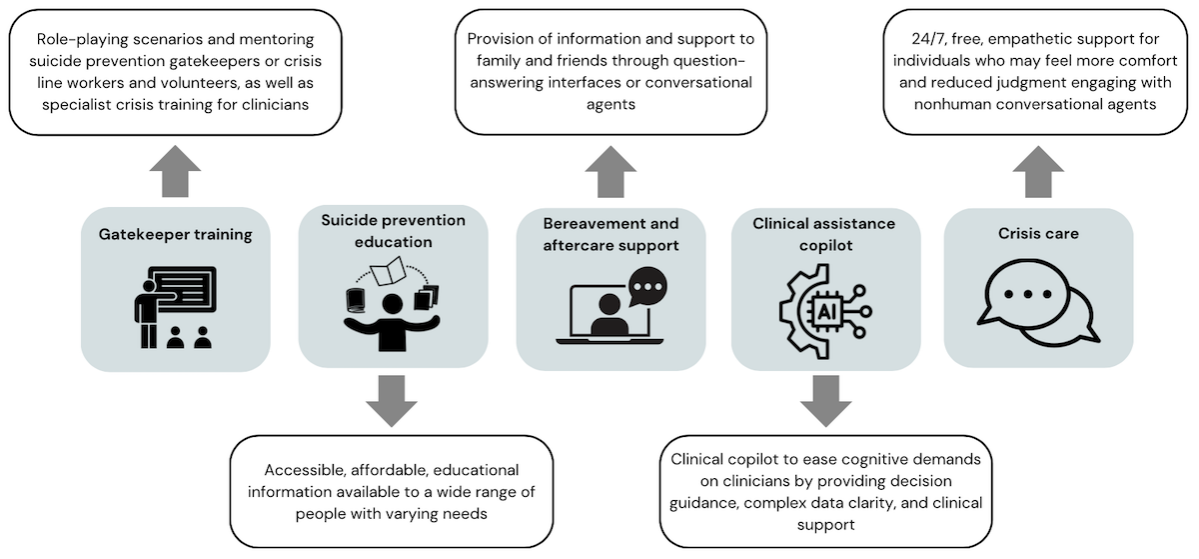
Potential future applications of large language models in the field of self-harm and suicide prevention. AI: artificial intelligence.

These training and education use cases should be moderated by human oversight and iterated with human feedback for continuous and rapid improvement. Future research should aim to develop models trained in this domain that can demonstrate reliability and effectiveness in providing training or educational outputs. The development of the underlying model in this use case has the potential to translate to alternative clinical applications while providing researchers with a greater understanding of the guardrails and parameters of operation that are required for safe deployment across suicide prevention applications (Figure 3).

### Ethical Considerations

Growing emphasis has been placed on the need for a clear and comprehensive ethical framework for integrating LLMs into mental health research and practice [111]. Hence, another aim of this review was to understand the ethical issues involved in the integration of LLMs into suicide prevention and self-harm research. Ethical considerations raised in the included studies focused on privacy and autonomy, bias, transparency and trust, and potential adverse impacts on the therapeutic relationship.

The integration of LLMs and social media data was noted as presenting a double-edged sword in terms of balancing privacy and confidentiality concerning the use of publicly available data with opportunities for suicide detection [55,75,82,83]. On the one hand, the vast repository of publicly available language-based data on social media platforms provides the potential for real time identification of individuals at risk. On the other hand, while such methods can be lifesaving, they bypass the consent traditionally required in research and clinical practice. User or poster consent was sought in only 1 (3%) of the 31 identified studies that used social media data for training their models. Users of social media sites are often not afforded the opportunity to opt out of such surveillance or analysis, nor are they made aware of the potential adverse consequences associated with having their data used in this manner (eg, the risk of data privacy breaches, as has happened with some LLMs) [112]. This contravenes the fundamental human right to privacy, recently underscored by European legislation prohibiting certain data-gathering practices related to health information by the algorithms of Facebook and other large social media companies [74,113]. This legislative approach is aligned with suggested practices to mitigate ethical concerns around privacy and confidentiality through the establishment of legal boundaries [82]. The use of social media and other data to train LLMs requires deep discussion to discern the regional, social, cultural, and temporal factors (among others) that influence ethical decisions around safe use of this data. An important ethical concern for the field of LLM-based suicide prevention research is how to balance the potential safety of users with their fundamental right to privacy. Inclusion of the lived experience perspective in this discussion is crucial if we are to arrive at a workable solution. Real-world clinical application of LLMs focused on detecting individuals at heightened suicide risk also raises critical ethical issues regarding the implications of false positives, if acted on by authorities, and the potential threats this poses to personal autonomy.

Bias is another important ethical consideration that was noted in the identified studies. In a recent review of AI algorithms applied to mental health, Straw Callison-Burch [114] found that significant biases exist with respect to religion, race, gender, nationality, sexuality, and age. These biases result from the expression of data (the manner in which the original data are presented) [115], the analysis of data (influenced by the contextual prelearnings of the model), and the interpretation of results (human annotation influenced by unconscious bias may produce model bias after training on that annotated data set) [114]. The presence of bias at any stage of model development risks creating tools that disadvantage certain groups of individuals [114]. Rigorous multimodel testing, comparison, and validation are required to isolate and mitigate inherent biases and thereby ensure unbiased performance across diverse populations. Understanding the specific factors that contribute to the effectiveness or limitations of a model also allows researchers to make informed decisions about how to optimize future training processes. This may involve collecting more diverse and representative data, implementing better preprocessing techniques, or fine-tuning model parameters.

Ethical issues of transparency and trust were also discussed in several of the included studies. In contrast to traditional machine learning algorithms, LLMs are opaque “black box” architectures with complex internal structures, which makes it difficult to understand and explain their decisions [67]. This is particularly challenging when hallucinations occur, with little recourse for discovering the source of the error. LLMs do not offer their “reasoning” unless prompted, and when prompted, they often fail to articulate how the provided information was retrieved, vetted, curated, or prepared. This lack of transparency is a fundamental flaw that hinders our ability to understand how these models operate, undermining their trustworthiness in health care applications [67]. Efforts are being made to distill this unknown “thinking” with the application of explainable AI methods. Of the 43 included studies, 2 (5%) [63,67] applied such methods in the form of Shapley additive explanations [116], local interpretable model-agnostic explanations [117], and Topic BERT [118]. The results indicate that these techniques can provide reasonable explainability for both short and long user-generated text and provide insights about data quality issues in training datasets [67]. However, current methods in this area fall somewhat short of delivering the transparency needed to establish total trust, limiting LLM deployment to contexts that restrict or protect against the potential for model hallucinations. Ongoing research into explainable AI techniques is critical in bridging this transparency gap to facilitate trust and enable real-world implementation.

Of the 43 included studies, 2 (5%) noted the client-clinician relationship as an ethical consideration: one in relation to LLM agents lacking human attributes [61] and the other noting a sense of distancing of the clinician from the individual, potentially fostering feelings of invalidation or insignificance [81]. At present, LLMs lack the authenticity and relational aspects required for modern mental health care [81]. Although LLMs can simulate empathy, it is important to consider the specific care needs of individuals experiencing suicidal ideation. If deployed incorrectly, LLM technologies have the potential to incite harm [119], requiring careful implementation to maintain safe practices. When consideration is given to the development of LLMs for use in suicide prevention, sound policy needs to be written to safeguard care recipients, reflecting the complexity of the relationships they have with traditional care providers [81]. In addition, stringent testing and oversight are required during the development and subsequent application of LLMs in the mental health domain [61]. While acknowledging the important ongoing role of human-led therapies, there is a clear need for further research into developing AI systems that can effectively integrate professionals into the loop. This would allow LLMs to serve as an aid in clinical settings, complementing rather than supplanting human practitioners.

### Limitations

Some limitations must be kept in mind when interpreting the findings of this scoping review. First, the categorization of machine learning technologies in the existing literature was not always clear, sometimes rendering it challenging to discern whether a study used an LLM or other machine learning model. It is possible that some relevant articles may have been excluded from the review. Second, due to the cross-disciplinary nature of the field and resulting publications, reporting detail varied between disciplines. Health-related publications provided more clinical and ethics-related information, whereas computer engineering publications were more likely to provide in-depth detail about the LLM model and training process. This made the synthesis and comparison of certain study attributes challenging; for example, data preparation and input methods were not sufficiently covered in the studies published in health-related journals to allow meaningful synthesis. Third, all data were extracted by a single author. Although data extraction was piloted by 2 authors to ensure consistency of approach at the outset, some data that were extracted were qualitative, potentially resulting in researcher bias; for example, data extraction related to ethical considerations such as privacy could be subject to bias due to researcher beliefs. Fourth, this review was conducted in an area of research that is currently experiencing significant growth and development; therefore, it only provides a time-stamped representation of the field. Finally, this review excluded studies using EHR data, given that EHR data may not be representative of individuals at risk of suicide because a large proportion of individuals experiencing suicidality are not in contact with formal mental health services [120]. However, excluding EHR studies reduced the number of included studies that focused on predicting suicide risk.

### Conclusions

LLMs represent a promising avenue for enhancing the scalability, accessibility, affordability, and personalization of tools in the field of suicide prevention and self-harm research; however, collaboration between computer science and mental health experts is essential to leverage the strengths of both disciplines effectively. This review identified a strong bias toward the use of BERT, with the potential for inherent biases indicating a pressing need for rigorous model comparison and testing, alongside the curation of diverse training datasets to mitigate model bias effectively. The growing use of generative LLM applications indicates promise for transformative applications in care, support, training, and education, such as improved crisis care and gatekeeper training methods, clinician copilot models, and improved educational practices. However, clinical accountability remains crucial to ensure the responsible use of LLMs in applications targeting suicide prevention. The identified ethical considerations underscore the need for clear governance via the establishment of policies and standards to guide the integration of LLMs into clinical use. LLMs have significant implications for self-harm and suicide prevention, underscoring the need to support continued research and development in this domain.

## Appendix

Multimedia Appendix 1 PRISMA-ScR (Preferred Reporting Items for Systematic Reviews and Meta-Analyses extension for Scoping Reviews) checklist.

Multimedia Appendix 2 Individual database search strings and data extraction template.

Multimedia Appendix 3 Table of included publications with extracted data.

## Abbreviations

AI: artificial intelligence
BERT: Bidirectional Encoder Representations from Transformers
EHR: electronic health record
FLAN: Fine-Tuned Language Net
GPT: generative pretrained transformer
LLM: large language model
PRISMA: Preferred Reporting Items for Systematic Reviews and Meta-Analyses
PRISMA-ScR: Preferred Reporting Items for Systematic Reviews and Meta-Analyses extension for Scoping Reviews

## References

[1] (2014). Preventing suicide: a global imperative. World Health Organization.

[2] Martinez-Ales G, Hernandez-Calle D, Khauli N, Keyes KM, Baca-Garcia E (2020). Why are suicide rates increasing in the United States? Towards a multilevel reimagination of suicide prevention. Behavioral Neurobiology of Suicide and Self Harm.

[3] Melia R, Francis K, Hickey E, Bogue J, Duggan J, O'Sullivan M, Young K (2020). Mobile health technology interventions for suicide prevention: systematic review. JMIR Mhealth Uhealth.

[4] Vaswani A, Shazeer N, Parmar N, Uszkoreit J, Jones L, Gomez AN, Kaiser L, Polosukhin I (2017). Attention is all you need. arXiv. Preprint posted online June 12, 2017.

[5] Yang R, Tan TF, Lu W, Thirunavukarasu AJ, Ting DS, Liu N (2023). Large language models in health care: development, applications, and challenges. Health Care Sci.

[6] Zhao WX, Zhou K, Li J, Tang T, Wang X, Hou Y, Min Y, Zhang B, Zhang J, Dong Z, Du Y, Yang C, hen Y (2023). A survey of large language models. arXiv. Preprint posted online March 31, 2023.

[7] Demszky D, Yang D, Yeager DS, Bryan CJ, Clapper M, Chandhok S, Eichstaedt JC, Hecht C, Jamieson J, Johnson M, Jones M, Krettek-Cobb D, Lai L, JonesMitchell N, Ong DC, Dweck CS, Gross JJ, Pennebaker JW (2023). Using large language models in psychology. Nat Rev Psychol.

[8] Gado S, Kempen R, Lingelbach K, Bipp T (2021). Artificial intelligence in psychology: how can we enable psychology students to accept and use artificial intelligence?. Psychol Learn Teach.

[9] He Y, Zhu Z, Zhang Y, Chen Q, Caverlee J (2020). Infusing disease knowledge into BERT for health question answering, medical inference and disease name recognition. arXiv. Preprint posted online October 8, 2020.

[10] Li C, Zhang Y, Weng Y, Wang B, Li Z (2023). Natural language processing applications for computer-aided diagnosis in oncology. Diagnostics (Basel).

[11] Wang J, Zhang G, Wang W, Zhang K, Sheng Y (2021). Cloud-based intelligent self-diagnosis and department recommendation service using Chinese medical BERT. J Cloud Comp.

[12] Omoregbe NA, Ndaman IO, Misra S, Abayomi-Alli OO, Damaševičius R (2020). Text messaging-based medical diagnosis using natural language processing and fuzzy logic. J Healthc Eng.

[13] Bala S, Keniston A, Burden M (2020). Patient perception of plain-language medical notes generated using artificial intelligence software: pilot mixed-methods study. JMIR Form Res.

[14] Peretz G, Taylor CB, Ruzek JI, Jefroykin S, Sadeh-Sharvit S (2023). Machine learning model to predict assignment of therapy homework in behavioral treatments: algorithm development and validation. JMIR Form Res.

[15] Tanana MJ, Soma CS, Kuo PB, Bertagnolli NM, Dembe A, Pace BT, Srikumar V, Atkins DC, Imel ZE (2021). How do you feel? Using natural language processing to automatically rate emotion in psychotherapy. Behav Res Methods.

[16] Shah RS, Holt F, Hayati SA, Agarwal A, Wang YC, Kraut RE, Yang D (2022). Modeling motivational interviewing strategies on an online peer-to-peer counseling platform. Proc ACM Hum Comput Interact.

[17] Allen NB, Nelson BW, Brent D, Auerbach RP (2019). Short-term prediction of suicidal thoughts and behaviors in adolescents: can recent developments in technology and computational science provide a breakthrough?. J Affect Disord.

[18] Coppersmith G, Leary R, Crutchley P, Fine A (2018). Natural language processing of social media as screening for suicide risk. Biomed Inform Insights.

[19] Peng C, Yang X, Chen A, Smith KE, PourNejatian N, Costa AB, Martin C, Flores MG, Zhang Y, Magoc T, Lipori G, Mitchell DA, Ospina NS, Ahmed MM, Hogan WR, Shenkman EA, Guo Y, Bian J, Wu Y (2023). A study of generative large language model for medical research and healthcare. NPJ Digit Med.

[20] Le Glaz A, Haralambous Y, Kim-Dufor DH, Lenca P, Billot R, Ryan TC, Marsh J, DeVylder J, Walter M, Berrouiguet S, Lemey C (2021). Machine learning and natural language processing in mental health: systematic review. J Med Internet Res.

[21] Vaidyam AN, Wisniewski H, Halamka JD, Kashavan MS, Torous JB (2019). Chatbots and conversational agents in mental health: a review of the psychiatric landscape. Can J Psychiatry.

[22] Xue J, Zhang B, Zhao Y, Zhang Q, Zheng C, Jiang J, Li H, Liu N, Li Z, Fu W, Peng Y, Logan J, Zhang J, Xiang X (2023). Evaluation of the current state of chatbots for digital health: scoping review. J Med Internet Res.

[23] Bendig E, Erb B, Schulze-Thuesing L, Baumeister H (2019). The next generation: chatbots in clinical psychology and psychotherapy to foster mental health – a scoping review. Verhaltenstherapie.

[24] Linthicum KP, Schafer KM, Ribeiro JD (2019). Machine learning in suicide science: applications and ethics. Behav Sci Law.

[25] Arowosegbe A, Oyelade T (2023). Application of natural language processing (NLP) in detecting and preventing suicide ideation: a systematic review. Int J Environ Res Public Health.

[26] Burke TA, Ammerman BA, Jacobucci R (2019). The use of machine learning in the study of suicidal and non-suicidal self-injurious thoughts and behaviors: a systematic review. J Affect Disord.

[27] Ji S, Pan S, Li X, Cambria E, Long G, Huang Z (2021). Suicidal ideation detection: a review of machine learning methods and applications. IEEE Trans Comput Soc Syst.

[28] D'Hotman D, Loh E (2020). AI enabled suicide prediction tools: a qualitative narrative review. BMJ Health Care Inform.

[29] Khan NZ, Javed MA (2022). Use of artificial intelligence-based strategies for assessing suicidal behavior and mental illness: a literature review. Cureus.

[30] Lejeune A, Le Glaz A, Perron P, Sebti J, Baca-Garcia E, Walter M, Lemey C, Berrouiguet S (2022). Artificial intelligence and suicide prevention: a systematic review. Eur Psychiatry.

[31] Bernert RA, Hilberg AM, Melia R, Kim JP, Shah NH, Abnousi F (2020). Artificial intelligence and suicide prevention: a systematic review of machine learning investigations. Int J Environ Res Public Health.

[32] Ophir Y, Tikochinski R, Brunstein Klomek A, Reichart R (2021). The Hitchhiker’s guide to computational linguistics in suicide prevention. Clin Psychol Sci.

[33] Pollock D, Peters MD, Khalil H, McInerney P, Alexander L, Tricco AC, Evans C, de Moraes ÉB, Godfrey CM, Pieper D, Saran A, Stern C, Munn Z (2023). Recommendations for the extraction, analysis, and presentation of results in scoping reviews. JBI Evid Synth.

[34] Peters MD, Godfrey C, McInerney P, Munn Z, Tricco A, Khalil H, Aromataris E, Lockwood C, Porritt K, Pilla B, Jordan Z (2020). Scoping reviews. JBI Manual for Evidence Synthesis.

[35] Tricco AC, Lillie E, Zarin W, O'Brien KK, Colquhoun H, Levac D, Moher D, Peters MD, Horsley T, Weeks L, Hempel S, Akl EA, Chang C, McGowan J, Stewart L, Hartling L, Aldcroft A, Wilson MG, Garritty C, Lewin S, Godfrey CM, Macdonald MT, Langlois EV, Soares-Weiser K, Moriarty J, Clifford T, Tunçalp Ö, Straus SE (2018). PRISMA extension for scoping reviews (PRISMA-ScR): checklist and explanation. Ann Intern Med.

[36] Peters MD, Marnie C, Tricco AC, Pollock D, Munn Z, Alexander L, McInerney P, Godfrey CM, Khalil H (2021). Updated methodological guidance for the conduct of scoping reviews. JBI Evid Implement.

[37] Holmes G, Whitton A, Tang B (2023). Leveraging language based AI to improve suicide prevention: a scoping review. Open Science Framework.

[38] Mohammad SM (2020). Examining citations of natural language processing literature. arXiv. Preprint posted online May 2, 2020.

[39] Tang S, Reily NM, Arena AF, Batterham PJ, Calear AL, Carter GL, Mackinnon AJ, Christensen H (2021). People who die by suicide without receiving mental health services: a systematic review. Front Public Health.

[40] Covidence review management. Covidence.

[41] Tang S, Werner-Seidler A, Torok M, Mackinnon AJ, Christensen H (2021). The relationship between screen time and mental health in young people: a systematic review of longitudinal studies. Clin Psychol Rev.

[42] Harvey D, Lobban F, Rayson P, Warner A, Jones S (2022). Natural language processing methods and bipolar disorder: scoping review. JMIR Ment Health.

[43] Amin MM, Cambria E, Schuller BW (2023). Can ChatGPT’s responses boost traditional natural language processing?. IEEE Intell Syst.

[44] Amin MM, Cambria E, Schuller BW (2023). Will affective computing emerge from foundation models and general artificial intelligence? A first evaluation of ChatGPT. IEEE Intell Syst.

[45] Ananthakrishnan G, Jayaraman AK, Trueman TE, Mitra SA, Murugappan A (2022). Suicidal intention detection in tweets using BERT-based transformers. Proceedings of the 2022 International Conference on Computing, Communication, and Intelligent Systems.

[46] Ascorbe P, Campos MS, Domínguez C, Heras J, Terroba-Reinares AR (2023). Towards a retrieval augmented generation system for information on suicide prevention. Proceedings of the 2023 IEEE EMBS Special Topic Conference on Data Science and Engineering in Healthcare, Medicine and Biology.

[47] Badian Y, Ophir Y, Tikochinski R, Calderon N, Klomek AB, Fruchter E, Reichart R (2023). Social media images can predict suicide risk using interpretable large language-vision models. J Clin Psychiatry.

[48] Broadbent M, Medina Grespan M, Axford K, Zhang X, Srikumar V, Kious B, Imel Z (2023). A machine learning approach to identifying suicide risk among text-based crisis counseling encounters. Front Psychiatry.

[49] Bucur AM, Cosma A, Dinu LP (2021). Early risk detection of pathological gambling, self-harm and depression using BERT. Proceedings of the 2021 Conference on Labs of the Evaluation Forum.

[50] Burkhardt HA, Ding X, Kerbrat A, Comtois KA, Cohen T (2023). From benchmark to bedside: transfer learning from social media to patient-provider text messages for suicide risk prediction. J Am Med Inform Assoc.

[51] Cao L, Zhang H, Feng L, Wei Z, Wang X, Li N, He X (2019). Latent suicide risk detection on microblog via suicide-oriented word embeddings and layered attention. Proceedings of the 2019 Conference on Empirical Methods in Natural Language Processing and the 9th International Joint Conference on Natural Language Processing.

[52] Cao L, Zhang H, Wang X, Feng L (2023). Learning users inner thoughts and emotion changes for social media based suicide risk detection. IEEE Trans Affective Comput.

[53] de Carvalho VC, Giacon B, Nascimento C, Nogueira BM (2020). Machine learning for suicidal ideation identification on Twitter for the Portuguese language. Intelligent Systems.

[54] Deshpande S, Warren J (2021). Self-harm detection for mental health chatbots. Stud Health Technol Inform.

[55] Diniz EJ, Fontenele JE, de Oliveira AC, Bastos VH, Teixeira S, Rabêlo RL, Calçada DB, Dos Santos RM, de Oliveira AK, Teles AS (2022). Boamente: a natural language processing-based digital phenotyping tool for smart monitoring of suicidal ideation. Healthcare (Basel).

[56] Dobbs MF, McGowan A, Selloni A, Bilgrami Z, Sarac C, Cotter M, Herrera SN, Cecchi GA, Goodman M, Corcoran CM, Srivastava A (2023). Linguistic correlates of suicidal ideation in youth at clinical high-risk for psychosis. Schizophr Res.

[57] Garg M (2023). Mental health analysis in social media posts: a survey. Arch Comput Methods Eng.

[58] Ghanadian H, Nejadgholi I, Osman HA (2023). ChatGPT for suicide risk assessment on social media: quantitative evaluation of model performance, potentials and limitations. arXiv. Preprint posted online June 15, 2023.

[59] Grimland M, Benatov J, Yeshayahu H, Izmaylov D, Segal A, Gal K, Levi-Belz Y (2024). Predicting suicide risk in real-time crisis hotline chats integrating machine learning with psychological factors: exploring the black box. Suicide Life Threat Behav.

[60] Haque F, Nur RU, Jahan SA, Mahmud Z, Shah FM (2020). A transformer based approach to detect suicidal ideation using pre-trained language models. Proceedings of the 23rd International Conference on Computer and Information Technology.

[61] Heston TF (2023). Safety of large language models in addressing depression. Cureus.

[62] Howard D, Maslej MM, Lee J, Ritchie J, Woollard G, French L (2020). Transfer learning for risk classification of social media posts: model evaluation study. J Med Internet Res.

[63] Islam MR, Sakib MK, Prome SA, Wang X, Ulhaq A, Sanin C (2023). Machine learning with explainability for suicide ideation detection from social media data. Proceedings of the 10th International Conference on Behavioural and Social Computing.

[64] Karapetian K, Jeon SM, Kwon JW, Suh YK (2023). Supervised relation extraction between suicide-related entities and drugs: development and usability study of an annotated PubMed corpus. J Med Internet Res.

[65] Lee DM, Moradi H (2022). Knowledge-infused dynamic embedding for predicting the severity of suicidal ideation in social media. Proceedings of the 2022 International Conference on Computational Science and Computational Intelligence.

[66] Levkovich I, Elyoseph Z (2023). Suicide risk assessments through the eyes of ChatGPT-3.5 versus ChatGPT-4: vignette study. JMIR Ment Health.

[67] Malhotra A, Jindal R (2024). XAI transformer based approach for interpreting depressed and suicidal user behavior on online social networks. Cogn Syst Res.

[68] Matero M, Idnani A, Son Y, Giorgi S, Vu HH, Zamani M, Limbachiya P, Guntuku SC, Schwartz HA (2019). Suicide risk assessment with multi-level dual-context language and BERT. Proceedings of the 6th Workshop on Computational Linguistics and Clinical Psychology.

[69] Metzler H, Baginski H, Niederkrotenthaler T, Garcia D (2022). Detecting potentially harmful and protective suicide-related content on twitter: machine learning approach. J Med Internet Res.

[70] Mezzi R, Yahyaoui A, Krir MW, Boulila W, Koubaa A (2022). Mental health intent recognition for Arabic-speaking patients using the mini international neuropsychiatric interview (MINI) and BERT model. Sensors (Basel).

[71] Naseem U, Khushi M, Kim J, Dunn AG (2024). Hybrid text representation for explainable suicide risk identification on social media. IEEE Trans Comput Soc Syst.

[72] Ravishankar TN, Kumar AK, Venkatesh J, Prabhu MR, Bhargavi VS, MuthamilSelvan S (2023). Empirical assessment and detection of suicide related posts in twitter using artificial intelligence enabled classification logic. Proceedings of the 2023 International Conference on Advances in Computing, Communication and Applied Informatics.

[73] Salmi S, Mérelle S, Gilissen R, van der Mei R, Bhulai S (2022). Detecting changes in help seeker conversations on a suicide prevention helpline during the COVID- 19 pandemic: in-depth analysis using encoder representations from transformers. BMC Public Health.

[74] Sawhney R, Joshi H, Nobles A, Shah RR (2021). Towards emotion- and time-aware classification of tweets to assist human moderation for suicide prevention. Proc Int AAAI Conf Web Soc Media.

[75] Schoene AM, Bojanić L, Nghiem MQ, Hunt IM, Ananiadou S, Schoene AM (2023). Classifying suicide-related content and emotions on Twitter using graph convolutional neural networks. IEEE Trans Affective Comput.

[76] Sharma N, Karwasra P (2023). Suicidal text detection on social media for suicide prevention using deep learning models. Proceedings of the TENCON 2023 - 2023 IEEE Region 10 Conference.

[77] Sheikh SA, Naidu H (2021). A novel robotics and MEMS artificial intelligence based train safety device. Proceedings of the 2nd International Conference on Smart Electronics and Communication.

[78] Soudi RB, Zaghloul MS, Badawy OM (2022). Framework for suicide detection from Arabic tweets using deep learning. Proceedings of the 32nd International Conference on Computer Theory and Applications.

[79] Wang S, Ning H, Huang X, Xiao Y, Zhang M, Yang EF, Sadahiro Y, Liu Y, Li Z, Hu T, Fu X, Li Z, Zeng Y (2023). Public surveillance of social media for suicide using advanced deep learning models in Japan: time series study from 2012 to 2022. J Med Internet Res.

[80] Wang S, Dang Y, Sun Z, Ding Y, Pathak J, Tao C, Xiao Y, Peng Y (2023). An NLP approach to identify SDoH-related circumstance and suicide crisis from death investigation narratives. J Am Med Inform Assoc.

[81] Woodnutt S, Allen C, Snowden J, Flynn M, Hall S, Libberton P, Purvis F (2024). Could artificial intelligence write mental health nursing care plans?. J Psychiatr Ment Health Nurs.

[82] Wu EL, Wu CY, Lee MB, Chu KC, Huang MS (2023). Development of Internet suicide message identification and the Monitoring-Tracking-Rescuing model in Taiwan. J Affect Disord.

[83] Xu X, Yao B, Dong Y, Gabriel S, Yu H, Hendler J, Ghassemi M, Dey AK, Wang D (2024). Mental-LLM: leveraging large language models for mental health prediction via online text data. Proceedings of the ACM on Interactive, Mobile, Wearable and Ubiquitous Technologies.

[84] Yen S, Chu K, Tsai P (2021). Prediction model of social network suicide ideation by small sample. Proceedings of the 22nd International Conference on Information Reuse and Integration for Data Science.

[85] Zhou W, Prater LC, Goldstein EV, Mooney SJ (2023). Identifying rare circumstances preceding female firearm suicides: validating a large language model approach. JMIR Ment Health.

[86] Chung HW, Hou L, Longpre S, Zoph B, Tay Y, Fedus W (2024). Scaling instruction-finetuned language models. J Mach Learn Res.

[87] Koya K, Chowdhury G (2020). Measuring impact of academic research in computer and information science on society. Proceedings of the 2020 2nd Asia Pacific Information Technology Conference.

[88] Chubb J, Cowling P, Reed D (2022). Speeding up to keep up: exploring the use of AI in the research process. AI Soc.

[89] Stade EC, Stirman SW, Ungar LH, Boland CL, Schwartz HA, Yaden DB, Sedoc J, DeRubeis RJ, Willer R, Eichstaedt JC (2024). Large language models could change the future of behavioral healthcare: a proposal for responsible development and evaluation. Npj Ment Health Res.

[90] Tsopra R, Fernandez X, Luchinat C, Alberghina L, Lehrach H, Vanoni M, Dreher F, Sezerman OU, Cuggia M, de Tayrac M, Miklasevics E, Itu LM, Geanta M, Ogilvie L, Godey F, Boldisor CN, Campillo-Gimenez B, Cioroboiu C, Ciusdel CF, Coman S, Hijano Cubelos O, Itu A, Lange B, Le Gallo M, Lespagnol A, Mauri G, Soykam HO, Rance B, Turano P, Tenori L, Vignoli A, Wierling C, Benhabiles N, Burgun A (2021). A framework for validating AI in precision medicine: considerations from the European ITFoC consortium. BMC Med Inform Decis Mak.

[91] Devlin J, Chang MW, Lee K, Toutanova K (2018). BERT: pre-training of deep bidirectional transformers for language understanding. arXiv. Preprint posted online October 11, 2018.

[92] Nadeem M, Bethke A, Reddy S (2020). StereoSet: measuring stereotypical bias in pretrained language models. arXiv. Preprint posted online April 20, 2020.

[93] Bender EM, Gebru T, McMillan-Major A, Shmitchell S (2021). On the dangers of stochastic parrots: can language models be too big? ????. Proceedings of the 2021 ACM Conference on Fairness, Accountability, and Transparency.

[94] Wu C, Lin W, Zhang X, Zhang Y, Xie W, Wang Y (2024). PMC-LLaMA: toward building open-source language models for medicine. J Am Med Inform Assoc.

[95] Toma A, Lawler PR, Ba J, Krishnan RG, Rubin BB, Wang B (2023). Clinical camel: an open-source expert-level medical language model with dialogue-based knowledge encoding. arXiv. Preprint posted online May 19, 2023.

[96] De Choudhury M, De S (2014). Mental health discourse on reddit: self-disclosure, social support, and anonymity. Proc Int AAAI Conf Web Soc Media.

[97] Chung NC, Dyer G, Brocki L (2023). Challenges of large language models for mental health counseling. arXiv. Preprint posted online November 23, 2023.

[98] Kirtley OJ, van Mens K, Hoogendoorn M, Kapur N, de Beurs D (2022). Translating promise into practice: a review of machine learning in suicide research and prevention. Lancet Psychiatry.

[99] Ahmad MA, Yaramis I, Roy TD (2023). Creating trustworthy llms: dealing with hallucinations in healthcare ai. arXiv. Preprint posted online September 26, 2023.

[100] Laka M, Carter D, Milazzo A, Merlin T (2022). Challenges and opportunities in implementing clinical decision support systems (CDSS) at scale: interviews with Australian policymakers. Health Policy Technol.

[101] Jung K, Kashyap S, Avati A, Harman S, Shaw H, Li R, Smith M, Shum K, Javitz J, Vetteth Y, Seto T, Bagley SC, Shah NH (2021). A framework for making predictive models useful in practice. J Am Med Inform Assoc.

[102] Shahsavar Y, Choudhury A (2023). User intentions to use ChatGPT for self-diagnosis and health-related purposes: cross-sectional survey study. JMIR Hum Factors.

[103] Lucas GM, Gratch J, King A, Morency LP (2014). It’s only a computer: virtual humans increase willingness to disclose. Comput Human Behav.

[104] Cascella M, Montomoli J, Bellini V, Bignami E (2023). Evaluating the feasibility of ChatGPT in healthcare: an analysis of multiple clinical and research scenarios. J Med Syst.

[105] Dergaa I, Fekih-Romdhane F, Hallit S, Loch AA, Glenn JM, Fessi MS, Ben Aissa M, Souissi N, Guelmami N, Swed S, El Omri A, Bragazzi NL, Ben Saad H (2023). ChatGPT is not ready yet for use in providing mental health assessment and interventions. Front Psychiatry.

[106] Acuña Caicedo RW, Gómez Soriano JM, Melgar Sasieta HA (2022). Bootstrapping semi-supervised annotation method for potential suicidal messages. Internet Interv.

[107] Hegerl U, Althaus D, Schmidtke A, Niklewski G (2006). The alliance against depression: 2-year evaluation of a community-based intervention to reduce suicidality. Psychol Med.

[108] Shand F, Torok M, Cockayne N, Batterham PJ, Calear AL, Mackinnon A, Martin D, Zbukvic I, Mok K, Chen N, McGillivray L, Phillips M, Cutler H, Draper B, Sara G, Christensen H (2020). Protocol for a stepped-wedge, cluster randomized controlled trial of the LifeSpan suicide prevention trial in four communities in New South Wales, Australia. Trials.

[109] Cross WF, Seaburn D, Gibbs D, Schmeelk-Cone K, White AM, Caine ED (2011). Does practice make perfect? A randomized control trial of behavioral rehearsal on suicide prevention gatekeeper skills. J Prim Prev.

[110] Holmes G, Clacy A, Hermens DF, Lagopoulos J (2021). The long-term efficacy of suicide prevention gatekeeper training: a systematic review. Arch Suicide Res.

[111] McKernan LC, Clayton EW, Walsh CG (2018). Protecting life while preserving liberty: ethical recommendations for suicide prevention with artificial intelligence. Front Psychiatry.

[112] Jang H A South Korean Chatbot shows just how sloppy tech companies can be with user data. The Slate Group LLC.

[113] The digital services act (DSA) - regulation (EU) 2022/2065. Digital Services Act.

[114] Straw I, Callison-Burch C (2020). Artificial Intelligence in mental health and the biases of language based models. PLoS One.

[115] Wongkoblap A, Vadillo MA, Curcin V (2017). Researching mental health disorders in the era of social media: systematic review. J Med Internet Res.

[116] Lundberg SM, Lee SI (2017). A unified approach to interpreting model predictions. Proceedings of the 31st International Conference on Neural Information Processing Systems.

[117] Ribeiro MT, Singh S, Guestrin C (2016). "Why should i trust you?" Explaining the predictions of any classifier. Proceedings of the 22nd ACM SIGKDD International Conference on Knowledge Discovery and Data Mining.

[118] Grootendorst M (2022). BERTopic: neural topic modeling with a class-based TF-IDF procedure. arXiv. Preprint posted online March 11, 2022.

[119] Arora A, Arora A (2023). The promise of large language models in health care. Lancet.

[120] Tang S, Reily NM, Arena AF, Sheanoda V, Han J, Draper B, Batterham PJ, Mackinnon AJ, Christensen H (2022). Predictors of not receiving mental health services among people at risk of suicide: a systematic review. J Affect Disord.

