# Supplementary material for: Applications of Large Language Models in the Field of Suicide Prevention: Scoping Review
Source: J Med Internet Res. 2025 Jan 23;27:e63126. doi: 10.2196/63126 (PMC11809463; doi:10.2196/63126)
Supplement: Multimedia Appendix 2 [file jmir_v27i1e63126_app2.docx]

**Multimedia Appendix 2**

Holmes G, Tang B, Gupta S, Venkatesh S, Christensen H, Whitton AE. Applications of Large Language Models in the Field of Suicide Prevention: A Scoping Review. Journal of Medical Internet Research. Accepted: Dec 10, 2024. DOI: 10.2196/63126

**Table S1.** Search Strings

| PubMed | ("suicide prevention"[MeSH Terms] OR "suicide"[MeSH Terms] OR "suicidal ideation"[MeSH Terms] OR "suicidal behav*"[Title/Abstract] OR "suicid*"[Text word] OR "suicide prevention"[Title/Abstract] OR "suicidality"[Title/Abstract] OR "suicide attempt*"[Title/Abstract] OR "suicidal attempt*"[Title/Abstract] OR "attempted suicid*"[Title/Abstract] OR "parasuicide"[Title/Abstract] OR "self-harm"[Title/Abstract] OR "self harm"[Title/Abstract] OR "DSH"[Title/Abstract] OR "NSSI"[Title/Abstract] OR "self-poison*"[Title/Abstract] OR "self poison*"[Title/Abstract] OR "self-injur*"[Title/Abstract] OR "self injur*"[Title/Abstract] OR "self-cut*"[Title/Abstract] OR "self cut*"[Title/Abstract] OR "self-mutilat*"[Title/Abstract] OR "self mutilat*"[Title/Abstract])  AND  ("natural language processing"[MeSH Terms] OR "natural language process*"[Text word] OR "NLP"[Text word] OR "large language model*"[Text word] OR "LLM"[Text word] OR "chatbot*"[Text word])  NOT  (“electronic health record*”[Text word] OR "electronic medical record*"[Text word] OR "digital health record*"[Text word] OR "digital medical record*"[Text word]) |
| --- | --- |
| EMBASE & PsycINFO (via Ovid) | (suicidal behaviour/ OR “suicidal behav*”.mp OR suicide/ OR “suicid*”.mp OR suicide prevention/ OR “suicide prevention” .mp OR “suicidality” .mp OR “suicide attempt*”.mp OR “suicidal attempt*”.mp OR “attempted suicid*”.mp OR “parasuicide”.mp OR “self-harm”.mp OR “self harm”.mp OR “DSH” .mp OR “NSSI” .mp OR “self-poison*”.mp OR "self poison*”.mp OR “self-injur*”.mp OR “self injur*”.mp OR “self-cut*”.mp OR “self cut*”.mp OR “self-mutilat*”.mp OR “self mutilat*”.mp)  AND  (natural language processing/ OR "natural language process*".mp OR “NLP”.mp OR “large language model*”.mp OR “LLM”.mp OR “chatbot*”.mp)  NOT  (“electronic health record*”.mp OR "electronic medical record*".mp OR "digital health record*".mp OR "digital medical record*".mp) |
| IEEE Xplore | ("Mesh_Terms":"suicide prevention" OR "Mesh_Terms":"suicide" OR "Mesh_Terms":"suicidal ideation" OR "Abstract":"suicidal behav*" OR "Abstract":"suicid*" OR "Abstract":"suicide prevention" OR "Abstract":"suicidality" OR "Abstract":"suicide attempt*" OR "Abstract":"suicidal attempt*" OR "Abstract":"attempted suicid*" OR "Abstract":"parasuicide" OR "Abstract":"self-harm" OR "Abstract":"self-harm" OR "Abstract":"DSH" OR "Abstract":"NSSI" OR "Abstract":"self injur*" OR "Abstract":"self injur*")  AND  ("Mesh_Terms":"natural language processing" OR "Full Text Only":"natural language process*" OR "Full Text Only":"NLP" OR "Full Text Only":"large language model*" OR "Full Text Only":"LLM" OR "Full Text Only":"chatbot" OR "Full Text Only":"chatbots")  NOT  ("Full Text Only":“electronic health record” OR "Full Text Only":“electronic health records” OR "Full Text Only":"electronic medical record" OR "Full Text Only":"electronic medical records" OR "Full Text Only":"digital health record" OR "Full Text Only":"digital health records" OR "Full Text Only":"digital medical record" OR "Full Text Only":"digital medical records”) |

Note: For IEEE Xplore search only Self-cut*, self cut*, self-poison*, self poison*, self-mutilat*, and self mutilat* are not included in IEEE Xplore search due to limitations on wildcard entries (9 max). For the same reason “chatbot*” was expanded to ["chatbot" OR "chatbots"] and “electronic health record*” was expanded to [“electronic health record” OR “electronic health records”] to ensure both variations of each term were captured whilst removing additional wildcards from the search.

**Table S2.** Data Extraction Template

| **Study Characteristics** | Title – Free text  Year of publication – Free text  Lead Author – Free text  Country of affiliation first author - Free text  Journal of publication – Free text  Journal of discipline – Single choice (health, computer engineering, other)  Funding Source – Free text (e.g., funding statement)  Suicide or self-harm related study – Tick box (suicide, self-harm)  Study area of focus – Single choice (i.e., Prediction, Identification/classification, Support, Education/training, Other [free text])  Aim of the study – Free text (what was the objective) |
| --- | --- |
| **Methods** | Data source – Free text (e.g., Reddit, Twitter/X, Survey, Crisis line etc.)  Which specific LLM was applied – Free text (e.g., GPT-4, RoBERTa, Alpaca, etc.)  Base LLM – Free text (list the original or base model)  LLM model training – Free text (describe model training if described)  LLM purpose – tick box (contextual understanding, generative, other)  LLM presentation – [avatar, audio, text, other?] (likely only applicable to generative cases)  Performance indicators – tick box (accuracy, precision, recall, F1, other [free text]) |
| **Outcomes** | Key findings – free text (description of results)  Clinical applications (if proposed by author) – free text (i.e., clinical/practical applications)  Ethical considerations (if proposed by author) – free text (not regarding study ethical approvals, but regarding the ethical implications of LLM application to humans. I.e., What ethical issues are identified by the author(s) as important for consideration? |
| **Reproducibility** | Is the dataset publicly available? Tick box (yes, no, other)  Is the code available for download? Tick box (yes, no, other) |
